# Measuring the Effects of Self-Awareness: Construction of the Self-Awareness Outcomes Questionnaire

**DOI:** 10.5964/ejop.v12i4.1178

**Published:** 2016-11-18

**Authors:** Anna Sutton

**Affiliations:** a Business School, Manchester Metropolitan University, Manchester, United Kingdom; The Maria Grzegorzewska University, Warsaw, Poland

**Keywords:** self-awareness, mindfulness, reflection, rumination, insight, work

## Abstract

Dispositional self-awareness is conceptualized in several different ways, including insight, reflection, rumination and mindfulness, with the latter in particular attracting extensive attention in recent research. While self-awareness is generally associated with positive psychological well-being, these different conceptualizations are also each associated with a range of unique outcomes. This two part, mixed methods study aimed to advance understanding of dispositional self-awareness by developing a questionnaire to measure its outcomes. In Study 1, expert focus groups categorized and extended an initial pool of potential items from previous research. In Study 2, these items were reduced to a 38 item self-report questionnaire with four factors representing three beneficial outcomes (reflective self-development, acceptance and proactivity) and one negative outcome (costs). Regression of these outcomes against self-awareness measures revealed that self-reflection and insight predicted beneficial outcomes, rumination predicted reduced benefits and increased costs, and mindfulness predicted both increased proactivity and costs. These studies help to refine the self-awareness concept by identifying the unique outcomes associated with the concepts of self-reflection, insight, reflection, rumination and mindfulness. It can be used in future studies to evaluate and develop awareness-raising techniques to maximize self-awareness benefits while minimizing related costs.

## Introduction

Self-awareness has long been seen by practitioners and researchers as both a primary means of alleviating psychological distress and the path of self-development for psychologically healthy individuals. Four decades ago, Fenigstein et al. wrote that “increased awareness of the self is both a tool and a goal” (Fenigstein, Scheier, & Buss, 1975, p. 522), while more recently an extensive review has demonstrated that different aspects of self-awareness, including mindfulness and rumination, mediate the impact of mindfulness-based interventions on mental health outcomes (Gu, Strauss, Bond, & Cavanagh, 2015). The importance of self-awareness goes beyond well-being and mental health to include substantial impacts on day-to-day functioning. It has important effects on performance, with reflection and mindfulness encouraging persistence with tasks despite performance-related stress (Feldman, Dunn, Stemke, Bell, & Greeson, 2014) and rumination related to interpersonal difficulties (Brinker, Chin, & Wilkinson, 2014).

While the number of studies demonstrating the importance of self-awareness continues to grow, there is not yet a comprehensive measure available to capture this range of effects and outcomes. Instead, it seems that each new study focuses on a different outcome, or that outcomes are investigated according to what is currently of wider interest in the psychological literature (as, for example, with the current interest in well-being). While this approach is certainly beneficial in establishing specific outcomes associated with self-awareness, it does leave the field somewhat fragmented. A single measure that could assess the whole range of potential outcomes of self-awareness would provide a dual benefit to researchers and practitioners. First, it would enable further theoretical differentiation of existing self-awareness concepts through a consideration of their differential impacts on individual lives. Second, it would provide an effective means for evaluating the potential changes brought about by awareness-building interventions. This paper reports on a two-part study to develop one such self-report questionnaire, the Self-Awareness Outcomes Questionnaire (SAOQ).

Self-awareness can be broadly defined as the extent to which people are consciously aware of their internal states and their interactions or relationships with others (see for example, Trapnell & Campbell, 1999; Trudeau & Reich, 1995). Viewed as an overarching theoretical construct, self-awareness is operationalized in different ways depending on the focus of the research. A distinction is often drawn, for example, between situational and dispositional self-awareness (Brown & Ryan, 2003), reflecting the different approaches of social psychologists and personality psychologists respectively.

Situational self-awareness is an automatic process by which we compare our current actions to our internalised standards, making changes where necessary to reduce inconsistency (Silvia & Duval, 2001). Dispositional self-awareness (also known as self-consciousness or self-attentiveness) in contrast, is the trait-like tendency for an individual to focus on and reflect on their own psychological processes and inner experiences as well as their relationships to others (Fenigstein et al., 1975). Fenigstein further distinguished between public and private self-consciousness, with the former being an awareness of how one appears to others (similar to the concept of self-monitoring) and the latter being awareness of and reflecting on one’s internal states. This distinction has, however, been challenged, with more recent authors providing evidence that public and private self-consciousness are domains for self-awareness rather than different types of self-awareness (Trapnell & Campbell, 1999).

There are several different measures of this dispositional self-attentiveness and the precise relationship between self-awareness and outcomes such as psychological well-being is dependent on which of these is used (Harrington & Loffredo, 2011). In the following sections, these different conceptualizations and measures are reviewed and their individual relationships with various outcomes are outlined.

The Self-Reflection and Insight Scale (SRIS: Grant, Franklin, & Langford, 2002) was developed as a measure of private self-consciousness which would assess internal state awareness (insight) separately from self-reflection. Self-reflection is defined as the extent to which an individual pays attention to and evaluates his/her internal states and behaviours, while insight is the clarity of understanding of these states and behaviours that the individual has. Grant et al. (2002) note that these abilities to monitor and evaluate are essential components of self-regulation and goal-directed behaviour. While self-reflection and insight are related to well-being, it is not a straightforward relationship. Insight is related to increased psychological well-being and cognitive flexibility, while self-reflection is associated with higher anxiety but lower depression. A so-called ‘self-absorption paradox’ seems to exist: higher self-attentiveness is associated with both better self-knowledge and increased psychological distress (Trapnell & Campbell, 1999).

This paradox was partially resolved when Trapnell and Campbell (1999) introduced a different conceptualization of dispositional self-attentiveness by relating self-awareness to the Big Five personality traits. Rumination, related to neuroticism, reflects a tendency to focus on negative self-perceptions and emotions. Reflection, on the other hand, is related to the openness to experience trait and represents a tendency to reflect objectively. The differential impact of these two forms of self-attentiveness have been demonstrated in many areas, including the interpersonal arena: rumination is associated with impaired interpersonal skills and increased negative affect while reflection is associated with improved interpersonal skills (Takano, Sakamoto, & Tanno, 2011).

A comparison of the SRIS and reflection / rumination conceptualizations of self-awareness demonstrated that Insight (from the SRIS scale) was the best predictor of six different dimensions of psychological well-being (Harrington & Loffredo, 2011). Rumination negatively predicted autonomy, mastery and self-acceptance while reflection positively predicted personal growth. This study further demonstrated how a focus on self-awareness outcomes can help to elucidate theoretical distinctions within the self-awareness concept.

A second and related conceptualization of self-awareness, namely mindfulness, has come under extensive scrutiny recently, with research generally indicating a positive relationship between mindfulness and well-being (Brown & Ryan, 2003) and mindfulness practice associated with long-term positive impacts on quality of life (Morgan, Graham, Hayes-Skelton, Orsillo, & Roemer, 2014). Mindfulness can be defined as attention to and awareness of the present moment which does not seek to react to or classify experience (Brown & Ryan, 2003). This element of a receptive attitude is helpful in differentiating between mindfulness and other aspects of self-awareness, as Brown and Ryan note when distinguishing between two unique modes of self-regulation: monitoring (represented by mindfulness) and controlling (represented by private self-attentiveness). Teasdale (1999) has suggested that this difference lies at the root of the differing effects of mindfulness and ruminative self-attentiveness, with the former being adaptive and the latter, due to its evaluative component, maladaptive.

The Mindful Attention Awareness Scale measures trait mindfulness and has been used to demonstrate wide ranging positive relationships between mindfulness and psychological health and functioning (Brown & Ryan, 2003). Despite this, there is emerging evidence that mindfulness is not always beneficial and can even, in certain circumstances, negatively impact on performance (Zhang, Ding, Li, & Wu, 2013). Indeed, in their seminal review, Brown, Ryan, and Creswell (2007) noted that being mindful could also potentially be harmful, for example by reducing the positive illusions that are associated with enhanced well-being. Yet this potentially harmful side of mindfulness is rarely explored in the profusion of literature about its beneficial effects. This study seeks to provide a holistic understanding of mindfulness and other dispositional aspects of self-awareness by identifying both the positive and negative outcomes associated with each.

Unless specifically referring to one of the individual conceptualizations reviewed above, this paper uses the broad definition of self-awareness as *a conscious awareness of one’s internal states and interactions with others*. A significant volume of research has elucidated the relationship of various conceptualizations of self-awareness with health-related variables (e.g. Ghasemipour, Robinson, & Ghorbani, 2013) and different psychological variables, particularly well-being (Brown et al., 2007). However, research on the more behavioural outcomes of increased self-awareness tends to be fragmented and focus on one or two outcomes at a time. If self-awareness is to be promoted as of direct value to individuals, organisations and society, it is important to assess the full range of potential outcomes. A comprehensive measure of the outcomes and effects of self-awareness would not only give researchers a concise way of evaluating the wide impact of self-awareness interventions but would also help to elaborate the nature of the relationships between different conceptualizations of self-awareness.

The following two-part study aimed to develop and evaluate one such measure, the Self-Awareness Outcomes Questionnaire. The SAOQ is an attempt to capture the range of effects that self-awareness has on an individual’s everyday life. Study 1 developed the conceptual framework and established face and content validity for the questionnaire. Study 2 established the factor structure, reliability and construct validity of the questionnaire, delineating the relationships between different measures of self-awareness and outcomes.

## Study 1: Conceptual Framework for the SAOQ

This study developed the content validity of the SAOQ questionnaire through identification, discussion and refinement of potential items with focus groups consisting of experts in the field of self-awareness.

### Method

Sutton et al. (2015) report on a longitudinal study of the effects of workshops designed to enhance self-awareness and identify four main themes and twelve contingent themes in the outcomes that participants report. Two of these twelve contingent themes were excluded from this study as not relevant: *‘no changes’* was excluded as the SAOQ is designed to measure only reported effects of self-awareness and *‘future development’* because the theme involved hopes or plans about future development which was not yet in evidence. An initial list of 61 self-awareness outcomes was developed based on the data from that study and these items were used as the basis for focus group discussions as follows.

#### Sample

Two expert focus groups were recruited through personal contacts, chosen to represent two different approaches to self-awareness development. The first consisted of four counsellors working within the person-centred (p-c) approach, who were also tutors on a p-c counselling course at a UK university. The development of self-awareness is a key aspect of the person-centred approach (Rogers, 1951) and these counsellors had many years of experience within the therapeutic and educational arenas.

The second focus group consisted of four therapists delivering first level psychological diagnosis and support as part of the NHS’s (National Health Service in the UK) Increasing Access to Psychological Therapy program. These therapists all had a nursing background and most of their therapeutic work used a CBT (cognitive behavioural therapy) approach. CBT also emphasises self-awareness on the part of both client and counsellor, though it is more focused on the client’s current situation than past experiences (NHS, 2012).

#### Procedure

The first focus group was conducted in a conference room on site at the university, while the second was at a training venue used previously by the therapists. Focus groups lasted approximately 90 minutes and were conducted by the author and a research assistant, with one leading the discussions and the other making detailed notes. The groups provided written informed consent and an audio recording of the discussions was made for later analysis. Participants were given a small voucher as thanks for their participation and this was provided at the beginning of the time so as not to pressure them into staying should they wish to leave the study early.

Participants were introduced to the study as an exploration of the effects of self-awareness on everyday life. The focus group discussions were initiated with a discussion about the participants’ own understanding of self-awareness and related concepts such as mindfulness, exploring how they as therapists viewed the concepts and drawing out their thoughts on how their clients understood them.

Each participant was then given cards with the potential questionnaire items on them (one per card) and asked to arrange them into themes or categories that made sense to them, and to give each theme a name. Participants were allowed to discuss their ideas with other members of the group as they did this, although most of them undertook the initial sorting individually.

Group discussions were then initiated to explore the themes that emerged for each person, why they chose these groupings and what differences the group noticed between different people’s understandings. Participants were then asked what effects of self-awareness they had noticed in their own, students’ or clients’ lives that were not recorded on the cards. Answers here were probed for both positive and negative effects.

### Results

The items were grouped into between three and six categories by the participants. While the individual names given to the categories varied among the participants, four general themes emerged: insight into and effect on myself, self-development and progress, interactions with and acceptance of others, and work-related outcomes.

Participants in both groups were able to identify further effects of improved self-awareness from their own experience and their observations of clients and students, including for *example I feel able to be different* and *I consciously choose to behave in certain ways rather than reacting instantly*. Another group of items - the perceived costs of self-awareness - was also identified by both groups (e.g. *I am more self-critical, I sometimes think “what have I been doing all these years?”*).

Besides these similarities between the two focus groups, participants in the second (CBT) group also focused on separating out cognitions / reflections from feelings, a reflection perhaps of the CBT approach to therapy. Although clearly an important part of CBT therapy, this distinction did not surface in the later analyses of questionnaire items in Study 2, where outcomes reflected the general themes agreed upon by both groups. Some participants in the CBT group also labelled items as positive or negative, and this distinction is developed somewhat in the interpretation of the subscales in Study 2, which identify both benefits and costs of self-awareness.

The final result of this study was a comprehensive list of 83 items representing the outcomes of self-awareness as reported by experts in the field and previous research evaluations of self-awareness training. Further, the themes and categories identified in this study provided context for interpretation of statistical results in Study 2.

## Study 2: Development of the SAOQ

Study 2 aimed to refine the items from the first study into a psychometrically sound self-report questionnaire that could be used to measure the outcomes self-awareness. In addition, Study 2 was designed to further explore the relationship between different self-awareness conceptualizations, including self-reflection, insight, reflection, rumination and mindfulness.

### Method

This study adopted a stratified sampling approach in order to recruit participants who could be theoretically expected to differ in the extent of their self-awareness. As discussed previously, counselling demands a high level of self-awareness in practitioners and counselling training courses emphasize the development of self-awareness in students. Counsellors and students on therapy-related courses were therefore expected to have higher levels of self-awareness than those employed in non-counselling fields or studying non-counselling courses. A sample of employed therapists and counsellors and a sample of students on counselling courses were therefore recruited. In order to provide a matched sample, participants were also recruited from two populations which were not engaged in self-awareness development: employees in non-therapy-related fields and non-counselling students. These participants were recruited via the author’s contacts in professional bodies and employing institution.

In summary, four different groups were sampled:

- Therapists and counsellors
- Post-graduate, part-time students on therapy-related courses
- Human Resources practitioners and Business School staff (i.e. employed but not in the therapy field)
- Post-graduate, part-time students on business courses.

The survey was advertised in professional newsletters, through personal contacts and on a forum for therapists-in-training, offering a small gift voucher as a thanks for their participation. Respondents to the advert were sent an email with a unique link to the online survey. In total, 215 participants were recruited.

#### Sample

The sample was 76% female with a mean age of 35.8 years (*SD* = 1.5), though it was skewed towards the younger end of the range, meaning that the mode age was 25 years. The majority of respondents were in work (77% full-time, 15% part-time) and 41% worked as therapists, either paid or voluntary. Students made up 47% of the sample, with an approximately even split between business-related and therapy-related courses.

#### Measures

The 83 items from Study 1 were reworded to allow the use of a frequency response scale and to ensure consistency in phrasing. They were arranged in random order and presented as a single questionnaire asking respondents to indicate how often they experienced each of the outcomes, with a five point frequency response scale from 1 (never) to 5 (almost always). In order to establish construct validity for the SAOQ, participants also completed the following measures:

The *Reflection Rumination Questionnaire* (Trapnell & Campbell, 1999) measures the extent to which a person tends to think about or reflect on self. The RRQ consists of 24 items measured on a 5 point Likert scale.

The *Mindful Attention Awareness Scale* (Brown & Ryan, 2003) measures trait mindfulness. The MAAS consists of 15 items measured on a 6 point frequency scale.

The *Self-Reflection and Insight Scale* (Grant et al., 2002) measures the tendency to reflect on the self and the extent to which individuals have insight into their own behaviour. The SRIS consists of 20 items measured on a 6 point scale.

*Self-awareness practices*: participants reported the frequency with which they engaged in a list of six mindfulness and self-awareness practices on a scale from 1 (never) to 7 (every day). These items were gathered from a literature review and the focus groups in Study 1 and included the following items: Meditation, Prayer, Mindfulness practice, “Talking therapy” (e.g. meeting with a counsellor), Writing a journal, Personal development group.

### Results

#### SAOQ Questionnaire Construction

The SAOQ data was tested for suitability for factor analysis. The Kaiser-Meyer-Olkin (KMO) measure of sampling adequacy was .74, indicating that a substantial proportion of the variance in the items was likely due to underlying factors. Bartlett’s test of sphericity was significant, indicating that the items were adequately related to each other. Factor analysis (alpha factoring with promax rotation) was conducted and the scree plot indicated that a five factor solution explaining 36% of the variance was most appropriate. Items which did not load clearly (above .4) on a single factor were excluded from further analysis and initial scales were constructed from the factor loadings of each item onto these five factors. Scale reliabilities were analysed and four scales with a reliability of α > .7 were retained.

Factor correlations indicated that an oblique transformation was not recommended (Tabachnick & Fidell, 2001). A final analysis was run on the remaining 38 items using alpha factoring extraction with promax rotation: alpha extraction is best for maximizing reliability and promax rotation is best for clarifying factors which are correlated with each other. The resulting 4 factor structure explained 44.4% of the variance. The pattern matrix is shown in Table 1 and, in combination with the qualitative analysis from Study 1, was interpreted to provide names for each of the subscales. In line with recommendations by Tabachnick and Fidell (2001), variables with loadings above.32 were interpreted. As this was an initial study to establish the wide range of different outcomes associated with self-awareness, it was considered better to be more rather than less inclusive of items as long as the reliability of the scale remained high. Items loading on more than one factor were interpreted within the factor with the highest loading.

**Table 1:** Pattern Matrix for SAOQ Items

SAOQ item	1	2	3	4
I "observe" myself	.82			
I have insight into myself	.73			
I look at why people act the way they do	.63			
I have learnt about myself and how I see the world	.63			
I am continuing to work on and develop myself	.60			
I focus on ways of amending my behaviour that would be useful	.60			
I feel generally positive about self-awareness	.53			
I reassess my own and others' responsibilities	.50			
I'm aware of my abilities and limitations	.42			
I am reflective	.41			
I am realistic about myself	.38	.35		
I have a good self-image		.77		
I feel on the whole very comfortable with the way I am		.75		
I have fun		.64		
I am consistent in different situations or with different people		.60		
I have compassion and acceptance for others		.57		
I interact well with colleagues or peers		.54		
I understand myself well	.33	.42		
I am confident		.42		-.32
I stop and think before judging		.41		
I understand my emotions		.40		
I am objective		.36		
I see my work life as something I have power to affect			.64	
I can “take a step back” from situations to understand them better			.61	
I am content with my work situation			.57	
I think about how my personality fits with my work role			.57	
I understand how I work within a team			.53	
I have changed the way I work	.31		.51	
I take control of my work			.47	
I recognize the stress and worry in my current work			.46	
I think about how as colleagues or peers we interact with each other	.35		.45	
I feel vulnerable				.67
I feel exposed				.66
I find making changes is difficult and scary				.65
I feel guilty for criticizing others				.56
I feel my emotions deeply				.51
I find it scary to try something new or step out of what I know.				.50
I have had to revisit difficult past experiences	.38			.42

*Note.* Loadings <.3 are not shown.

The reflective self-development subscale (RSD, 11 items, α = .87) contains items representing the development of continuous attention to the self, with a focus on conscious, reflective and balanced learning. The acceptance of self and others subscale (Acceptance, 11 items, α = .83) represents outcomes including a positive self-image and confidence as well as a deeper understanding of others. The proactive at work subscale (Proactive at Work, 9 items, α = .81) consists of items specifically related to the outcomes of self-awareness in the workplace and represents an objective and proactive approach to dealing with work. The final subscale represents the emotional costs of self-awareness (Emotional Costs, 7 items, α = .77) and includes items representing the potential negative emotional impacts of being more aware of oneself, such as guilt, fear, vulnerability and fear.

#### Relationship of SAOQ With Self-Awareness

Table 2 gives the descriptive statistics for the scales used in this study. The correlational analysis indicated that those who ruminate more are more likely to experience the costs of self-awareness and less likely to be accepting of themselves and others. Those who reflect more are more likely to experience reflective self-development outcomes but also more likely to experience the costs. When measured with the SRIS-SR scale, self-reflection was also associated with increased proactivity at work. Interestingly, mindfulness (MAAS) only showed associations with negative outcomes: decreased acceptance of self and others and increased costs. The negative association with acceptance runs counter to much published research and may indicate that this sample was somewhat unique in that a higher score on the MAAS was not associated a non-evaluative state of mind. Uniquely, the SRIS-Insight scale was associated positively with all the benefits of self-awareness (RSD, acceptance and proactivity subscales) and negatively with the emotional costs. Finally, increased engagement in self-awareness practices is associated with increased reflective self-development and acceptance, but also increased costs.

Multiple regression analyses were conducted to establish how different conceptualizations of self-awareness were related to the outcomes measured in the SAOQ (Table 3). Self-awareness variables were entered as a block in individual analyses to predict each of the SAOQ subscales. Overall, self-awareness measures explained between 12% and 41% of the variance in each of the four subscales and different conceptualizations of self-awareness predicted different subscale outcomes.

RSD was positively predicted by SRIS-SR and self-awareness practices. The Acceptance subscale was predicted positively by SRIS-Insight and self-awareness practices, and negatively by rumination. The Proactive at Work subscale was positively predicted by mindfulness and SRIS-SR and negatively by rumination. Finally, emotional costs were positively predicted by rumination, mindfulness and self-awareness practices.

Between them, the SRIS subscales positively predicted the three benefits subscales. Mindfulness predicted proactivity but also increased costs. Rumination predicted a reduction in experienced self-awareness benefits (acceptance and proactivity) and an increase in costs. Engagement in self-awareness practices predicted both benefits (reflective self-development and proactivity) and emotional costs.

**Table 2:** Descriptive Statistics and Correlations for all Scales

Scale	*M*	*SD*	1	2	3	4	5	6	7	8	9	10
1. SAOQ RSD	3.94	.58	(.87)									
2. SAOQ Acc	3.85	.52	.50**	(.83)								
3. SAOQ Pro	3.74	.68	.42**	.37**	(.81)							
4. SAOQ Cost	3.10	.67	.16*	-.18**	-.06	(.77)						
5. RRQ Rumination	3.34	.78	.05	-.40**	-.12	.51**	(.92)					
6. RRQ Reflection	3.46	.72	.50**	-.02	.14	.25**	.25**	(.91)				
7. MAAS	3.06	.74	-.07	-.32**	.03	.44**	.47**	.09	(.88)			
8. SRIS Insight	4.10	.79	.16*	.35**	.16*	-.38**	-.42**	-.05	-.51**	(.84)		
9. SRIS-SR	4.41	.89	.57**	-.01	.21**	.25**	.30**	.77**	.01	.05	(.93)	
10. Self-awareness practices	1.94	.98	.43**	.24**	.14	.27**	.05	.41**	.04	-.03	.37**	(.75)

*Note.* Alpha reliabilities in brackets. SAOQ = Self-Awareness Outcomes Questionnaire; RSD = Reflective self-development; Acc = Acceptance; Pro = Proactive at work; Cost = Emotional Costs; RRQ = Reflection Rumination Questionnaire; MAAS = Mindful Attention Awareness Scale; SRIS = Self-Reflection and Insight Scale; SR = Self-Reflection.

*p < .05 (2-tailed). **p < .01 (2-tailed).

**Table 3:** Predictors of Self-Awareness Outcomes (SAOQ Subscales): Regression Analyses

	RSD	Acc	Pro	Cost
**Model *R^2^***	.41	.27	.12	.38
***F* (6,188)**	21.60***	11.78***	4.16**	19.45***
Standardized betas				
RRQ-Rum	-.06	-.27**	-.23**	.31***
RRQ-Ref	.11	-.05	-.09	-.02
MAAS	-.01	-.11	.21*	.22**
SRIS-In	.13	.19*	.15	-.14
SRIS-SR	.41***	0	.31**	.10
Practices	.24***	.28***	.06	.21**

*p < .05. **p < .01. ***p < .001.

*Note.* RSD = Reflective self-development; Acc = Acceptance; Pro = Proactive at work; Cost = Emotional Costs.

#### SAOQ in Different Groups

As expected, the subsamples differed in their levels of self-awareness. T-tests demonstrated that students on therapy-related courses were more reflective (RRQ-reflection: *t*(81) = -3.3, *p* < .01, *d* = .74; SRIS-SR: *t*(79) = -2.5, *p* < .05, *d* = .56) and engaged in more self-awareness practices (*t*(76) = -5.16, *p* < .001, *d* = 1.18) than students on business-related courses. Those respondents actively engaged in providing therapy (whether paid or voluntary) scored lower on rumination (*t*(204) = 3.6, *p* < .001, *d* = .5) and engaged in more self-awareness practices (*t*(193) = -3.3, *p* < .001, *d* = .48) than non-therapists.

Scores on the SAOQ demonstrated that these differences in self-awareness were reflected in different reported outcomes for each group as well. Therapy students reported higher costs (*t*(78) = -4.08, *p* < .001, *d* = .92) than business students, while therapists reported more reflective self-development (*t*(197) = -2.93, *p* < .01, *d* = .42) and proactivity at work (*t*(199) = -2.13, *p* < .05, *d* = .30) than non-therapists. All these group differences showed medium to large effect sizes, using the ranges recommended by Cohen.

## Discussion

This study aimed to extend our understanding of the self-awareness concept, particularly given the current interest in mindfulness, and to establish a questionnaire that could be used as a single measure of the wide range of outcomes of self-awareness in the general population.

Four factors emerged from the statistical analysis in the second study which showed substantial similarity to the themes identified by focus group experts in the first study: reflective self-development (a combination of the ‘insight into myself’ and ‘self-development’ themes identified in the qualitative study), acceptance of self and others, proactivity at work, and emotional costs. The correspondence between these qualitatively and quantitatively derived factors provides between-method triangulation support (Denzin, 1970) for the structure of the SAOQ, indicating that it captures both statistically-sound and qualitatively meaningful outcomes of self-awareness.

One important finding to note is the relatively high correlations between the RSD outcomes scale and the two measures of reflective self-awareness (RRQ-Ref and SRIS-SR). While correlations above .5 indicate a strong relationship between the variables, there is still a substantial amount of unshared variance, indicating that the self-awareness scales and the outcomes scales are assessing different concepts. In addition, there is a conceptual difference to bear in mind. The RRQ and SRIS scales were developed as measures of *trait* self-awareness, while the SAOQ-RSD scale was developed from participants’ and experts’ reports of the effects of self-awareness, specifically the further *development* of reflective self-awareness. As this was a cross-sectional study, it is not possible to identify whether trait reflection is the cause of greater experience of reflective self-development or whether reflective self-development results in an increase in trait reflection. However, previous work has indicated that engagement in self-awareness training can indeed have some impact on trait reflection (Sutton, Williams, & Allinson, 2015) and it seems likely that reflection and development interact closely: increased reflection results in increased self-development, which in turn promotes higher levels of trait reflection in the long term.

The three distinct types of self-awareness benefits identified here lend support to Brown et al.’s (2007) review of the beneficial effects of mindfulness and extend their applicability to other conceptualizations of self-awareness. For example, the acceptance scale reflects the improved social interaction quality that Brown et al identified. Addressing their concern about delineating the processes by which mindfulness brings about these outcomes, the RSD scale may be useful in identifying how self-awareness is an ongoing, iterative process, where some of the outcomes are also part of the developmental process itself (e.g. *I am continuing to work on and develop myself*). It is plausible that enhancing self-awareness creates a virtuous cycle resulting in increasing benefits beyond the initial impact of any intervention.

The SAOQ also identifies a distinct group of costs associated with self-awareness. This subscale deserves special consideration as it clarifies the specific negative outcomes that Brown et al suggested were in need of further exploration. In addition, it helps to explore the self-absorption paradox which Trapnell and Campbell (1999) related to a ruminative or reflective tendency in self-attentiveness. In this study, rumination and mindfulness both predicted an increase in the experienced emotional costs of self-awareness. However, while rumination also predicted a decrease in proactivity at work and acceptance, mindfulness predicted increased proactivity at work. This is consistent with suggestions that mindfulness may be a multi-faceted concept and that the non-judging facet may be particularly important in determining the experienced benefits (Peters, Eisenlohr-Moul, Upton, & Baer, 2013). It also reinforces the growing literature sounding a note of caution about the impact of mindfulness on everyday life (e.g. Zhang et al., 2013) by highlighting a significant link with negative outcomes.

In contrast to the mixed results of mindfulness, the SRIS scales (Grant et al., 2002) predicted solely positive outcomes. Insight predicted acceptance and self-reflection predicted both reflective self-development and proactivity. Previous work has found the insight scale to be most predictive of positive well-being outcomes (Harrington & Loffredo, 2011), and this research contributes to our understanding of the positive impact of the self-reflection scale by taking account of factors beyond psychological well-being. While the self-attentiveness paradox highlights a certain increase in some negative well-being measures (Grant et al., 2002), it seems that this negative impact does not carry through to wider outcome measures. Further research comparing the impact of self-awareness interventions using both the SAOQ and measures of psychological well-being, or assessing potential suppressor variables (Simsek, 2013), could help to explore this issue.

For professionals engaged in developing self-awareness, whether tutors on therapy courses, therapists in a mental health context, or consultants developing mindfulness-related skills in an occupational context, this study identifies important implications for practice. First, the SAOQ enables practitioners and researchers to measure the specific effects of self-awareness interventions. Second, this study highlights the need for awareness of the potential costs of developing self-awareness. Given the current high level of interest in and promotion of mindfulness in many different contexts, an awareness of these emotional costs is important in providing people with a balanced view of the effects that mindfulness is likely to have.

In the wider business context, the SAOQ can provide a unique and comprehensive method for measuring outcomes of real-world relevance. For example, continuing professional development (CPD) is an essential component of expertise in many professions, from healthcare to Human Resource Management, and professional bodies are increasingly emphasising the centrality of reflective ability in this process. The SAOQ’s reflective self-development subscale provides a useful way of measuring the extent to which professionals are improving in their ability to reflect and develop and to evaluate training designed to help them in this skill development. In addition, the acceptance subscale measures outcomes around improved teamwork and communication, which are highly valued and sought-after outcomes in most organisational contexts. Overall, the SAOQ provides organisations with a way to capture the important changes that can be expected as a result of any self-awareness training that is undertaken.

### Limitations

Although the SAOQ was developed with a stratified sample, representative of the different levels of self-awareness in the population, it remains for future research to test the questionnaire with a more diverse sample. Studies like this may identify differing effects of self-awareness dependent on context. Given the relationship of self-reflective tendencies to personality traits (Trapnell & Campbell, 1999), future research could also investigate the extent to which personality may impact on the likelihood of experiencing different self-awareness outcomes.

In addition, the SAOQ was developed as a measure of the effects of self-awareness which included work-based outcomes: future research exploring the effects of self-awareness in a range of occupational groups and cultures would help to extend our knowledge of the impact of self-awareness in different work contexts. While the proactivity at work scale is particularly useful for evaluating interventions in the workplace, it is less useful for non-working populations. Work to develop an equivalent scale containing items unrelated to work but still demonstrating proactive changes would expand the utility of this questionnaire.

Finally, the questionnaire construction in Study 2 utilized a cross-sectional approach to relate the measured outcomes to self-awareness levels, which is of course limited in its predictive value. However, the design of the whole study, from the initial development of items from a longitudinal study of self-awareness training, to their development through expert focus groups on the effects of self-awareness, to the triangulation of findings through quantitative and qualitative approaches, can give a fair degree of confidence in the direction of influence from self-awareness to outcomes. Further longitudinal research would help to confirm the utility of targeted self-awareness training in developing these outcomes.

### Conclusion

By developing a measure of the outcomes of self-awareness, this study has contributed to extending our understanding of the self-awareness concept and its effects in everyday life. The SAOQ identifies the main impacts of self-awareness on people’s day-to-day lives and provides initial evidence of the outcomes associated with the practice of common mindfulness and self-awareness techniques. It can be used in future studies of the comparative effect of these techniques in order to identify ways of improving self-awareness that can enhance reflective self-development, acceptance and proactivity while minimizing related emotional costs.

## References

[r1] Brinker J. K. Chin Z. H. Wilkinson R. (2014). Ruminative thinking style and the MMPI-2-RF. Personality and Individual Differences, 66, 102–105. doi:. 10.1016/j.paid.2014.03.001

[r2] Brown K. W. Ryan R. M. Creswell J. D. (2007). Mindfulness: Theoretical foundations and evidence for its salutary effects. Psychological Inquiry, 18(4), 211–237. 10.1080/10478400701598298

[r3] Brown K. W. Ryan R. M. (2003). The benefits of being present: Mindfulness and its role in psychological well-being. Journal of Personality and Social Psychology, 84(4), 822–848. 10.1037/0022-3514.84.4.822 12703651

[r4] Denzin, N. K. (1970). *The research act: A theoretical introduction to sociological methods*. Chicago, IL, USA: Aldine Publishers.

[r5] Feldman G. Dunn E. Stemke C. Bell K. Greeson J. (2014). Mindfulness and rumination as predictors of persistence with a distress tolerance task. Personality and Individual Differences, 56, 154–158. doi:. 10.1016/j.paid.2013.08.040 24298196 PMC3843486

[r6] Fenigstein A. Scheier M. F. Buss A. H. (1975). Public and private self-consciousness: Assessment and theory. Journal of Counselling and Clinical Psychology, 43(4), 522–527. 10.1037/h0076760

[r7] Ghasemipour Y. Robinson J. A. Ghorbani N. (2013). Mindfulness and integrative self-knowledge: Relationships with health-related variables. International Journal of Psychology, 48(6), 1030–1037. doi:. 10.1080/00207594.2013.763948 23410242

[r8] Grant A. M. Franklin J. Langford P. (2002). The Self-Reflection and Insight Scale: A new measure of private self-consciousness. Social Behavior and Personality, 30(8), 821–835. 10.2224/sbp.2002.30.8.821

[r9] Gu J. Strauss C. Bond R. Cavanagh K. (2015). How do mindfulness-based cognitive therapy and mindfulness-based stress reduction improve mental health and wellbeing? A systematic review and meta-analysis of mediation studies. Clinical Psychology Review, 37, 1–12. 10.1016/j.cpr.2015.01.006 25689576

[r10] Harrington R. Loffredo D. A. (2011). Insight, rumination, and self-reflection as predictors of well-being. The Journal of Psychology, 145(1), 39–57. doi:. 10.1080/00223980.2010.528072 21290929

[r11] Morgan L. P. K. Graham J. R. Hayes-Skelton S. A. Orsillo S. M. Roemer L. (2014). Relationships between amount of post-intervention mindfulness practice and follow-up outcome variables in an acceptance-based behavior therapy for Generalized Anxiety Disorder: The importance of informal practice. Journal of Contextual Behavioral Science, 3(3), 173–178. doi:. 10.1016/j.jcbs.2014.05.001 25328862 PMC4196425

[r12] NHS. (2014). *Cognitive behavioural therapy (CBT).* Retrieved from http://www.nhs.uk/conditions/Cognitive-behavioural-therapy/Pages/Introduction.aspx

[r13] Peters J. R. Eisenlohr-Moul T. A. Upton B. T. Baer R. A. (2013). Nonjudgment as a moderator of the relationship between present-centered awareness and borderline features: Synergistic interactions in mindfulness assessment. Personality and Individual Differences, 55(1), 24–28. doi:. 10.1016/j.paid.2013.01.021

[r14] Rogers, C. R. (1951). *Client-centered therapy: Its current practice, implications, and theory.* Boston, MA, USA: Houghton Mifflin Company.

[r15] Silvia P. J. Duval T. S. (2001). Objective self-awareness theory: Recent progress and enduring problems. Personality and Social Psychology Review, 5(3), 230–241. 10.1207/S15327957PSPR0503_4

[r16] Simsek O. F. (2013). Self-absorption paradox is not a paradox: Illuminating the dark side of self-reflection. International Journal of Psychology, 48(6), 1109–1121. 10.1080/00207594.2013.778414 23560421

[r17] Sutton A. Williams H. M. Allinson C. W. (2015). A longitudinal, mixed method evaluation of self-awareness training in the workplace. European Journal of Training and Development, 39(7), 610–627. doi:. 10.1108/EJTD-04-2015-0031

[r18] Tabachnick, B. G., & Fidell, L. S. (2001). *Using multivariate statistics* (4th ed.). London, United Kingdom: Allyn and Bacon.

[r19] Takano K. Sakamoto S. Tanno Y. (2011). Ruminative and reflective forms of self-focus: Their relationships with interpersonal skills and emotional reactivity under interpersonal stress. Personality and Individual Differences, 51(4), 515–520. doi:. 10.1016/j.paid.2011.05.010

[r20] Teasdale J. D. (1999). Emotional processing, three modes of mind and the prevention of relapse in depression. Behaviour Research and Therapy, 37(Suppl. 1), S53–S77. doi:. 10.1016/S0005-7967(99)00050-9 10402696

[r21] Trapnell P. D. Campbell J. D. (1999). Private self-consciousness and the Five Factor Model of Personality: Distinguishing rumination from reflection. Journal of Personality and Social Psychology, 76(2), 284–304. 10.1037/0022-3514.76.2.284 10074710

[r22] Trudeau K. J. Reich R. (1995). Correlates of psychological mindedness. Personality and Individual Differences, 19(5), 699–704. 10.1016/0191-8869(95)00110-R

[r23] Zhang J. Ding W. Li Y. Wu C. (2013). Task complexity matters: The influence of trait mindfulness on task and safety performance of nuclear power plant operators. Personality and Individual Differences, 55(4), 433–439. doi:. 10.1016/j.paid.2013.04.004

